# Whole-animal genome-wide RNAi screen identifies networks regulating male germline stem cells in *Drosophila*

**DOI:** 10.1038/ncomms12149

**Published:** 2016-08-03

**Authors:** Ying Liu, Qinglan Ge, Brian Chan, Hanhan Liu, Shree Ram Singh, Jacob Manley, Jae Lee, Ann Marie Weideman, Gerald Hou, Steven X. Hou

**Affiliations:** 1 Basic Research Laboratory, National Cancer Institute at Frederick, National Institutes of Health, 1050 Boyles Street, Building 560, Room 12-70, Frederick, Maryland 21702, USA

## Abstract

Stem cells are regulated both intrinsically and externally, including by signals from the local environment and distant organs. To identify genes and pathways that regulate stem-cell fates in the whole organism, we perform a genome-wide transgenic RNAi screen through ubiquitous gene knockdowns, focusing on regulators of adult *Drosophila* testis germline stem cells (GSCs). Here we identify 530 genes that regulate GSC maintenance and differentiation. Of these, we further knock down 113 selected genes using cell-type-specific Gal4s and find that more than half were external regulators, that is, from the local microenvironment or more distal sources. Some genes, for example, *versatile* (*vers*), encoding a heterochromatin protein, regulates GSC fates differentially in different cell types and through multiple pathways. We also find that mitosis/cytokinesis proteins are especially important for male GSC maintenance. Our findings provide valuable insights and resources for studying stem cell regulation at the organismal level.

## Abstract (web summary)

Both intrinsic and external signals regulate stem cell fate. Here, the authors perform a genome-wide RNAi screen to identify regulators of testis germline stem cells (GSC) in *Drosophila*, finding more than half of the genes were external regulators and heterochromatin/cytokinesis proteins mediated GSC fate.

## Graphical abstract

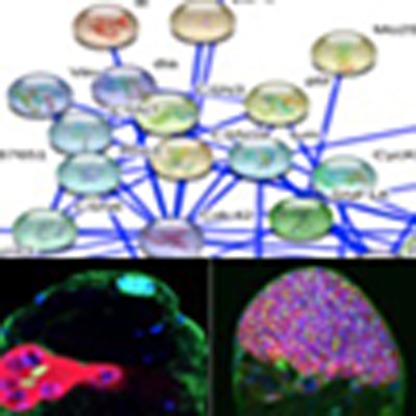

Stem cells are crucial components of many animal tissues and organs. Germline stem cells (GSCs) are unique because of transmitting genomic information to future generations. GSCs in *Drosophila* testis provide an excellent opportunity to study the defining mechanisms of stem cells at the cellular and molecular levels (reviewed in refs 1, 2). GSCs in the *Drosophila* testis reside at the apical tip of the testis that maintain spermatogenesis^1^,^2^. Each GSC is enclosed by two somatic cyst stem cells (CySCs). A recent study suggests that CySCs encystment promotes GSCs abscission during GSC division due to inhibition of cytokinesis^3^. Both the GSCs and CySCs are attached to a cluster of postmitotic somatic cells called the hub^4^, via cadherin-mediated cell adhesion^5^,^6^. The somatic hub serves as a niche, which expresses the signalling ligand for the *Drosophila* Janus kinase–signal transducer and activator of transcription (JAK-STAT) pathway, Unpaired (Upd). Upd instructs the JAK-STAT pathway in neighbouring GSCs and CySCs to regulate their self-renewal. Besides the JAK-STAT pathway, there are other regulatory pathways that also control the fate of GSCs and CySCs at the testis niche^1^,^3^,^7^,^8^,^9^,^10^,^11^,^12^,^13^,^14^,^15^,^16^,^17^,^18^,^19^,^20^.

In CySCs, JAK-STAT signalling and its putative targets (such as such as *zinc-finger homeodomain-1 (zfh-1)* and *chinmo*) both act intrinsically to regulate CySC self-renewal and maintenance, and non-autonomously regulate the self-renewal of adjacent GSCs^21^,^22^. Epidermal growth factor receptor (EGFR)-Ras/Raf signalling also acts both intrinsically to regulate CySCs and non-autonomously to regulate adjacent GSCs, but has the opposite effects of JAK-STAT signalling^23^,^24^,^25^. In addition, a Polycomb group component, Enhancer of Zeste, acts intrinsically in CySCs to control CySC activity and identity, and non-autonomously to prevent adjacent GSC ectopic self-renewal and conversion to a somatic cell identity^26^. However, it is unclear how these pathways are interconnected in CySCs to both control CySCs intrinsically and regulate GSCs non-autonomously.

In GSCs, the mother (old) centrosome always anchored close to the niche during GSC division, whereas the daughter centrosome moves to the reverse side of the cell, congregating a mitotic spindle perpendicular towards the hub^27^,^28^. GSCs actively monitor centrosome orientation through a centrosome orientation checkpoint (COC)^8^,^27^,^29^. The centrosome component Centrosomin and the cell polarity kinase Par-1 are essential components of the COC. The polarity protein Bazooka (Baz) provides a platform for the correct centrosome orientation and the COC monitors centrosome orientation by controlling the Baz–centrosome association. GSCs with COC defects enter mitosis without the correct centrosome orientation, leading to misoriented spindles and symmetric stem cell divisions^30^,^31^. Further, the mother centriole nucleates excess microtubules compared with the daughter centriole that may promote asymmetric Apc2 (Adenomatosis polyposis coli 2) delivery to the cortex where GCSs contact the hub^28^. In addition, at the cortex, Apc2 and E-cadherin together anchor the spindles of mitotic GSCs perpendicular to the hub^31^. As a result, only one daughter cell is directly connected to the hub and receives JAK-STAT signal to sustain its stem cell identity, whereas the other daughter cell, at the opposite end of the mitotic spindle, experiences a much weaker signal and begins differentiation. Recently, we found that nucleoporin and spindle matrix protein Tpr/Mtor (megator) regulate GSC asymmetric division and maintenance^16^. Loss of *Mtor* function affects expression and localization of Apc2 and E-cadherin. Further, we found that Mtor is essential for proper centrosome orientation, mitotic spindle formation and chromosome segregation. Our results suggest that nuclear matrix-SAC (spindle assembly checkpoint) axis controls maintenance and asymmetric division of GSC through the Mtor-Mps1 (monopolar spindle 1)/Mad2 (mitotic arrest deficient 2) pathway^16^.

Recent developments in genome-wide RNA interference (RNAi) techniques in *Drosophila* have enabled the knockdown of nearly complete sets of genes involved in cellular processes in living animals^32^,^33^,^34^. In addition, genome-wide RNAi screens have been performed to identify regulatory networks in several types of stem cells^35^,^36^,^37^,^38^. However, stem cells are regulated not only intrinsically but also by extracellular cues from the local environment^39^; these previous screens focused only on identifying intrinsic regulators. Furthermore, signals from distant organs also regulate stem cell/progenitor maintenance^40^,^41^ and organ–organ communications are very important in regulating organismal growth and ageing^42^,^43^,^44^.

The fruit fly *Drosophila* uniquely enables the systematic study of stem cell biology at the organismal level. To comprehensively identify genes and pathways that regulate GSC fates from different cell types in the whole organism, we perform a genome-wide transgenic RNAi screen through ubiquitous knockdowns of genes in adult *Drosophila* and then examine male GSC defects. Here we identify 530 genes whose RNAi-mediated knockdown affects stem cell maintenance and differentiation. Of these, we further knock down selected genes using cell-type-specific Gal4s and find that more than half are external regulators of GSC fate that originate from either the local microenvironment or distant organs. Moreover, we identify genes that can differentially regulate GSC fates from different cell types and through multiple pathways. Our data provide valuable insight and a useful resource for studying stem cell regulation at the organismal level.

## Results

### Developing the high-throughput screen

To systematically analyse the function of individual genes in the male GSC, we screened the existing Vienna *Drosophila* RNAi Center (VDRC) and the Bloomington *Drosophila* Stock Center (BDSC) collection of long double-stranded RNA (dsRNA) and short small hairpin RNA (shRNA) lines. The RNAi methodology has definite restrictions^32^,^33^,^34^. First, the P-element-based UAS-hairpin constructs incorporate haphazardly into the genome and the level of hairpin expression is influenced by its chromosomal location. Second, the RNA level is reduced only to a variable degree by the RNAi-mediated knockdown that, in some cases, results in negligible effect. Third, null mutations of a large number of non-essential genes do not cause a phenotype (FlyBase).

**Figure 1:**
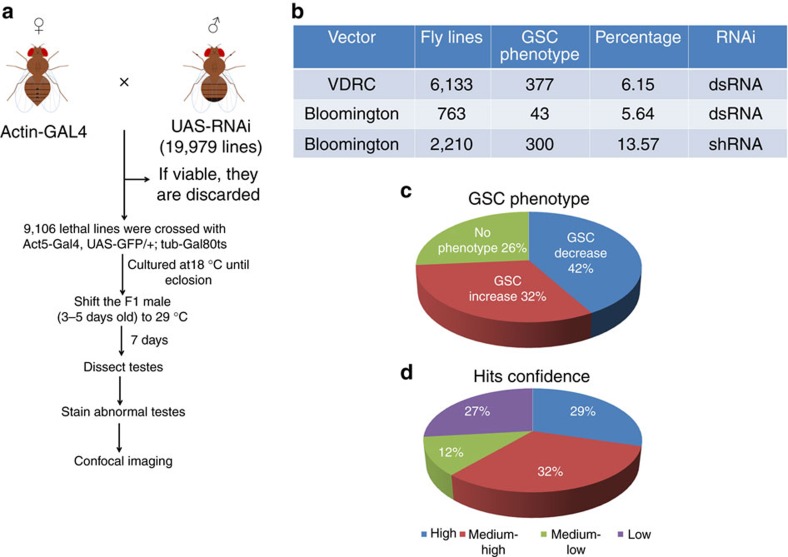
Transgenic RNAi screen. (**a**) Workflow of the GSC RNAi screen. (**b**) Pie chart summarizing the screen results. Fly lines shown in the table are those that gave a lethal phenotype when crossed with *Act-Gal4*. (**c**) Pie chart summarizing phenotype observed in all the 720 lines. *Act*^ts^>*RNAi* male flies were dissected after 7 days at 29 °C. Their testes were stained with antibodies (vasa, 1B1 and Arm) and analysed by confocal microscopy. The phenotypes were divided into two major categories (GSC decrease and increase). Percentage of fly lines showing no phenotypes are also shown in the chart. (**d**) Confidence of 530 genes identified from the screen. High-confidence genes were identified by two or more independent RNAi lines. Medium-high-confidence genes were identified by one RNAi line, but they shared a complex with high-confidence hits. Medium-low-confidence genes were identified by one RNAi line, but they shared a complex with other low-confidence hits. Low-confidence hits were identified by one RNAi only.

To reduce the overall false-negative rate and conduct an efficient screen, we first performed a pilot experiment in which we selected 1,000 RNAi lines at random. Each of these lines was crossed in duplicate to actin GAL4 driver fly line (*Act5-Gal4)* and to *Act5-Gal4, UAS-GFP/+; tub-Gal80*^ts^/*+*drivers. The progeny from the cross with *Act5-Gal4* were screened for lethality and any visible adult phenotype. Those from the cross with *Act5-Gal4, UAS-GFP/+; tub-Gal80*^ts^/*+* were scored for GSC phenotypes. We found that 90.4% of the RNAi lines with GSC phenotypes were lethal in the cross with *Act5-Gal4.* Thus, in the following screen, we first crossed all the RNAi lines with *Act5-Gal4* to test for lethality and then crossed only the lethal lines with *Act5-Gal4, UAS-GFP/+; tub-Gal80*^ts^/*+* to screen for GSC phenotypes (Fig. 1a).

### Genome-wide RNAi screen for male GSC using *Act-Gal4*

To identify candidate genes involved in male GSC regulation, we performed a systematic RNAi screen *in vivo*. We ubiquitously expressed *UAS-RNAi* in adult *Drosophila* using the *Act5-Gal4, UAS-GFP/+;tub-Gal80*^ts^/*+* driver^45^. Crossing of virgin females (*Act5-GAL4*, *UAS-GFP/+;tub-Gal80*^ts^/+) with two to three males from *UAS-RNAi* fly lines were set at 18 °C. Male adult flies from F1 generation were shifted to 29 °C, to induce RNAi expression. After 7 days, testes of F1 males were dissected and examined for phenotypes, and only abnormal testes were stained with antibodies and examined under confocal microscopy (Fig. 1a).

GSCs in the adult *Drosophila* testis are regulated both intrinsically and externally by signals from the hub and CySCs (reviewed in ref. 1). In addition, trachea-derived decapentaplegic (Dpp) regulates stem cells in the adult *Drosophila* midgut^40^ and olfactory receptor neurons regulate blood progenitor maintenance by controlling *γ*-aminobutyric acid secretion^41^. Furthermore, organ–organ communication plays important roles in regulating organism growth and ageing^42^,^43^,^44^,^46^. Thus, signals from both the local environment and distant organs could be important for GSC regulation and much of the complexity of stem cell regulation would be lost if we limited our screen to one cell type. Therefore, we first performed the genome-wide transgenic RNAi screen by the ubiquitous knockdown of genes with *Act-Gal4* and then characterized the functions of selected genes in individual cells with cell-type-specific Gal4 (such as germ cells specific driver, *Nanos (Nos)-Gal4* or CySC and early cyst-cell driver (*c587-Gal4*). This is similar to first performing a classical ethyl methanesulfonate- or P-element-mediated screen and then characterizing the functions of selected genes in individual cells by mosaic clonal analysis.

In total, we screened 19,979 transgenic lines of either dsRNA or shRNA from both the VDRC and the BDSC (Fig. 1b and Supplementary Data 1), representing 13,019 of the 14,139 protein-coding genes (92%) in release 5.7 of the *Drosophila* genome^47^. Among the 19,979 transgenic lines tested, 9,106 (45.6%) lines, corresponding to 6,873 genes, were lethal, once expressed by the *Act5-Gal4* driver.

**Figure 2:**
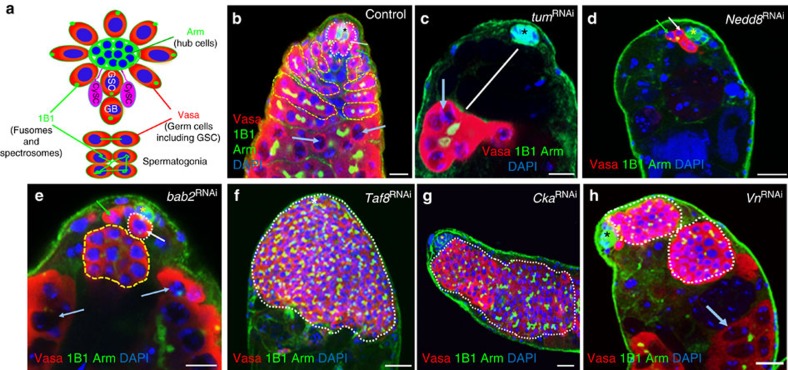
Representative RNAi screen phenotypes using *Act*^*ts*^*-Gal4*. (**a**) Diagram of GSC lineage and marker expression. GSCs and GBs have round spectrosomes (green dots) and spermatogonia contain branched fusomes (green branched lines). GSCs are enclosed by two CySCs (pink). (**b**) Wild-type (WT) control. (**c**) Knockdown of *tum* resulted in GSC loss and premature differentiation into spermatogonial cells (white arrow). (**d**) Knockdown of *Nedd8* resulted in GSC loss and death of differentiated cells. (**e**) Knockdown of *bab2* (*bric a brac 2*) resulted in GSC loss and GSCs that directly differentiated into spermatogonial cells (yellow dotted line) and spermatocytes (light blue arrows). (**f**) Knockdown of *Taf8* resulted in GSC differentiation defects. (**g**) Knockdown of *Cka* (*Connector of kinase to AP-1*) resulted in GSC overproliferation at the expense of terminal differentiation. (**h**) Knockdown of *Vein* (*Vn)* resulted in GSC differentiation defects. Testes of the indicated flies were dissected, stained with antibodies to Vasa (red, marks all germ cells including GSCs), Arm (green, hub cells at the apex), 1B1 (green, marks round spectrosomes and branched fusomes) and DAPI (blue, marks the nuclei), and analysed by confocal microscopy. Asterisks indicate hub cells. White arrows near hub cells indicate GSC and green arrows indicate GBs (in **b**,**d**,**e**), and light blue arrows (**b**,**c**,**e**,**h**) indicate spermatocytes. White dotted lines indicate GSCs (near hub in **a**) and GSC-like cells (in **f**–**h**). Scale bars, 10 μm.

**Figure 3:**
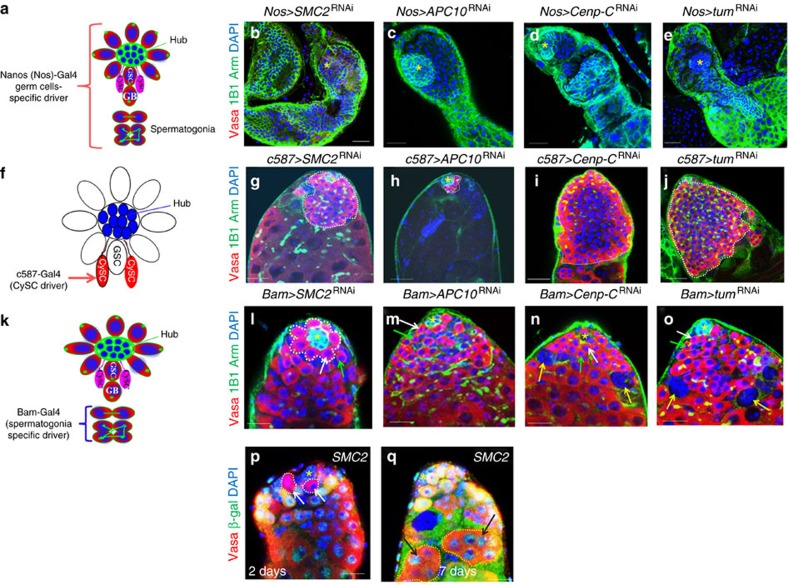
Cell-type-specific knockdowns of mitotic/cytokinesis genes. (**a**) A schematic diagram showing expression pattern of *Nos-Gal4* in adult testis. Knockdown of *Smc2* (**b**), *APC10* (**c**), *Cenp-C (Centromeric protein-C)* (**d**) and *tum* (**e**) in germ cells (*Nos-Gal4*) resulted in dramatic GSC loss. (**f**) A schematic diagram showing expression pattern of *c587-Gal4* in adult testis. Knockdown of *Smc2* (**g**), *Cenp-C* (**i**) and *tum* (**j**) in CySC (*c587-Gal4*) resulted in GSC differentiation defects (white dotted lines, **g**,**i**,**j**); however, knockdown of *APC10* (**h**) in CySCs resulted in GSC loss. (**k**) A schematic diagram showing expression pattern of *Bam-Gal4* in adult testis. Knockdown of *Smc2* (**l**) and *APC10* (**m**) *Cenp-C* (**n**) and *tum* (**o**) in spermatogonial cells (*Bam-Gal4*) resulted in normal (**l**,**m**) or polyploid (**n**,**o**, yellow arrows) cells. (**p**,**q**) *FRT*^42D^*-SMC2*^f06842^ mosaic clones of GSCs 2 days (**p**, white dotted lines with white arrows, β-galactosidase (green) negative) and 7 days (**q**, yellow dotted lines with black arrows, β-galactosidase (green) negative) after clonal induction (ACI). Arm-negative GSC clones were detected in 4 out of 8 testes 2 days ACI but in 0 out of 20 testes 7 days ACI. Therefore, SMC2 is intrinsically required for GSC maintenance. Testes of the indicated flies were dissected, stained with antibodies to Vasa (red, marks all germ cells including GSCs), Arm (green, hub cells at the apex), 1B1 (green, marks round spectrosomes and branched fusomes) and DAPI (blue, marks the nuclei) in **b**–**o**, and analysed by confocal microscopy. White arrows near hub cells indicate GSC and green arrows indicate GBs (in **l**–**o**). Asterisks indicate hub cells. The flies were cultured at 29 °C for 7 days in **b**–**o**. Scale bars, 10 μm.

**Figure 4:**
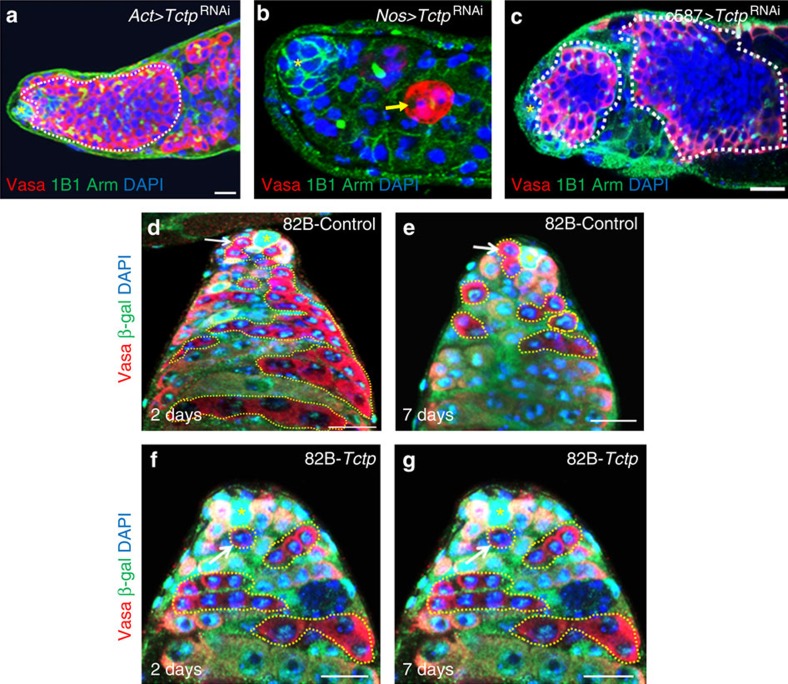
*TCTP* differentially regulate GSCs from different cells. (**a**) Knockdown of *Tctp* using *Act-Gal4* resulted in GSC differentiation defects. (**b**) Knockdown of *Tctp* using *Nos-Gal4* resulted in GSC loss. (**c**) Knockdown of *Tctp* using *c587-Gal4* resulted in GSC differentiation defects. *FRT*^82B^*-piM* mosaic clones of GSCs 2 days (**d**) and 7 days (**e**) ACI. *FRT*^82B^*-Tctp*^h5p^ mosaic clones of GSCs 2 days (**f**) and 7 days (**g**) ACI. In control flies, Arm-lacZ-negative GSC clones were detected in 33 out of 37 testes 2 days ACI and in 51 out of 64 testes 7 days ACI; in *Tctp*^h5p^ flies, Arm-lacZ-negative GSC clones were detected in 37 out of 53 testes 2 days ACI and in 8 out of 63 testes 7 days ACI. Thus, *Tctp* is intrinsically required for GSC maintenance. Testes of the indicated flies were dissected, stained with antibodies to Vasa (red, germ cells including GSCS), β-galactosidase (green) and DAPI (blue, marks the nuclei) in **d**–**g**, and Vasa (red), Arm (green, hub cells at the apex), 1B1 (green, marks round spectrosomes and branched fusomes) and DAPI (blue, marks the nuclei) in **a**–**c**, and analysed by confocal microscopy. The flies were cultured at 29 °C for 7 days in **a**–**c**. White arrows near hub cells indicate a GSC clone (**d**–**f**) and yellow dotted lines in **d**–**g** indicate GSC clones. White dotted lines in **a**,**c** indicate GSC-like cells. A yellow arrow in **b** indicates a remaining germ cell. Asterisks indicate hub cells. Scale bars, 10 μm.

**Figure 5:**
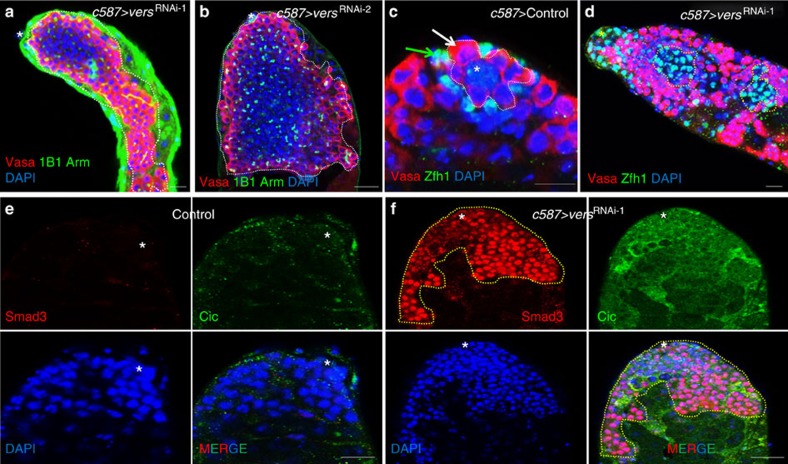
Vers interacts with EGFR and Dpp to regulate GSC and CySC. (**a**,**b**) Knockdown of *vers* in CySC lineage *c587-Gal4*) caused GSC differentiation defects. (**c**) Wild-type (control) testis. (**d**) Knockdown of *vers* in CySC lineage (*c587-Gal4*) caused CySC expansion. (**e**) Wild-type (control) testis. (**f**) Knockdown of *vers* in CySC lineage (*c587-Gal4*) activates both EGFR and Dpp signalling. Testes of the indicated flies were dissected, stained with antibodies to Vasa (red), Arm (green, hub cells at the apex) and 1B1 (green, marks round spectrosomes and branched fusomes) in **a**,**b**, Vasa (red) and Zfh1 (green, marks CySCs) in **c**,**d**, Smad3 (red) and Cic (green) in **e**,**f**, and DAPI (blue, marks the nuclei), and analysed by confocal microscopy. White dotted lines in **a**,**b** indicate GSC-like cells. White arrow near hub cells indicates a GSC and green arrow indicates a CySC (**c**). Yellow dotted lines in **f** indicate Smad3-positive cells. Asterisks indicate hub cells. The flies were cultured at 29 °C for 7 days. Scale bars, 10 μm.

We then expressed the 9,106 transgenic RNAi lines with *Act5-Gal4, UAS-GFP/+; tub-Gal80*^ts^/*+* (Fig. 1a) and analysed 5–10 flies for each line for GSC phenotypes (Fig. 1c). A total of 720 promising lines scored from the first-round screen, corresponding to 530 genes (Fig. 1b,d and Supplementary Data 2), were repeatedly screened and stained with molecular markers to confirm the phenotypes (Figs 2, 3, 4, 5).

Through a BLAST search for all of the other available lines in the VDRC and BDSC, which were generated from independent or mini-overlapping sequences for all 530 genes, we found only 319 dsRNA lines (319 genes) have other available lines in the VDRC and BDSC. Among these 319 genes, the phenotypes of 150 genes were confirmed by two or more independent lines; the other 169 genes had either no other independent line or other independent line but lacked a phenotype, possibly due to low knockdown efficiency (Supplementary Data 1). The phenotypes of a number of genes, including the following four, were confirmed using only two independent lines, even if more than two independent lines were obtained: CG1098/Madm (MLF1-adaptor molecule)—BL316644 (GSC loss)/V27346 (GSC loss)/BL42529 (no phenotype), CG7467/osa—BL31266 (GSC loss)/V7810 (GSC loss)/BL38258 (no phenotype), CG8553/SelD (Selenide)—V35959 (GSC loss)/BL29553 (GSC loss)/V100820 (no phenotype) and CG32183/Ccn—V38208 (GSC loss)/BL31715 (GSC loss)/V101518 (no phenotype). This result showed that some independent lines had low knockdown efficiency. Among 211 shRNA lines, the phenotypes of 10 were confirmed in two or more independent lines. We did not further confirm the other 201 shRNA lines, because lines generated from shRNAs usually have better efficiency and fewer off-targets than dsRNA lines^34^.

### Quality evaluation

Six lines of evidence suggest that our screen identified GSC regulators with high confidence. First, it is known that the JAK-STAT and EGFR-Ras/Raf signal transduction pathways are major pathways that regulate male GSCs, often with opposite effects^21^,^22^,^23^,^24^,^25^. We identified four positive regulators in the JAK-STAT pathway whose knockdown resulted in GSC loss, nine positive regulators in the EGFR-Ras/Raf pathway whose knockdown caused GSC differentiation defects and four other components of the EGFR pathway, whose knockdown resulted in GSC loss (Supplementary Data 2). Second, many of the other genes found in our screen were previously shown to regulate GSC functions (Supplementary Data 2). Third, many of the identified hits (385 genes), whose products are components of protein complexes, showed high phenotypic similarity (Fig. 1c and Supplementary Data 2 and 3). Fourth, of 530 genes identified, 319 were positive targets from dsRNA lines, among which we verified 150 genes by at least two or more independent lines (Supplementary Data 1 and 2); the other 211 genes were positive targets from shRNA lines, which are known to have good efficiency and few off-targets^34^. We also verified some of the genes identified in the RNAi screen by mutant clone analysis (Figs 3p,q and 4d,g). Furthermore, many of the GSC-specific genes were lethal in the primary screen, but did not show GSC phenotypes. Fifth, among the remaining 141 low-confidence genes, 78 (78/141=55%) were knocked down with shRNA lines and 41 (41/141=29%) were identified in at least 1 of 3 previous stem cell RNAi screens (neuroblast^35^, female GSC^36^ and intestinal stem cell (ISC)^37^; Supplementary Data 4). Finally, we showed efficient knockdown of a select set of genes by quantitative PCR analysis (Supplementary Data 5).

### Genes whose knockdown resulted in GSC loss

In the wild-type testes, five to nine GSCs reside at the apical niche (Fig. 2a,b). RNAi-mediated knockdowns of genes required for GSC self-renewal and maintenance resulted in GSC loss. We identified 300 such genes (Figs 1c and 2c–e, and Supplementary Data 2).

### JAK-STAT pathway

The JAK-STAT signalling pathway was the first one reported to regulate stem cell maintenance in the *Drosophil*a testis^24^,^48^. In the testis, the ligand Upd is expressed in hub cells and activates JAK-STAT signalling in adjacent stem cells for the maintenance of both GSCs and CySCs. When JAK-STAT signalling is depleted from testis cells, both GSCs and CySCs are completely lost. In contrast, when the signalling is amplified by overexpressing *upd* throughout the testis apex or by overexpressing a constitutively activated form of JAK kinase, *hopscotch tumorous lethal* (*hop*^Tum-l^) in somatic cells, the testes are filled with self-renewing GSCs interspersed among the CySCs^1^,^15^,^21^. In this screen, we found that knockdown of the receptor gene *dome (domeless)*, the *Drosophila stat* gene *stat92E* and two new positive regulators in the pathway, *CG6946/glo (glorund)*^49^ and *CG31716/Cnot4* (*Cnot 4 homologue)*^50^, resulted in GSC loss. Surprisingly, the knockdown of *Drosophila PIAS (protein inhibitor of activated STAT-1)* gene (*dPIAS)* also resulted in GSC loss. dPIAS was initially identified as a negative regulator of the JAK-STAT pathway and later found in the heterochromatin protein complex involved in epigenetic modification^51^,^52^,^53^. Thus, dPIAS may regulate GSCs through a JAK-STAT-independent pathway.

### Mitosis/cytokinesis genes

In mammalian cells after anaphase onset, the mitotic spindle is completely reorganized into an array of interdigitating and antiparallel microtubules, known as the central spindle. Many signalling and cytoskeletal proteins cooperate to assemble and organize the central spindle. These include the microtubule-associated protein regulatory of cytokinesis 1 (PRC1, *Drosophila* Fascetto/Feo), the chromosomal passenger complex and several kinesin-like motors: KIF4A (*Drosophila* kinesin-like protein at 3A/Klp3A), KIF14 (*Drosophila* Nebbish/Neb), KIF20A (*Drosophila* Subito) and KIF23 (*Drosophila* Pavarotti/Pav). KIF4A transports PRC1 to the spindle midzone, KIF14 interacts with PRC1 and KIF23 is important for bundling central spindle microtubules to form a protein complex known as centralspindlin. Centralspindlin is a conserved heterotetramer composed of KIF23/Pav and RacGAP1/MgcRacGAP (*Drosophila* Tumbleweed/Tum) dimers. The central spindle plays a key role in cleavage furrow formation. In addition, the formation of an active zone of RhoA at the equatorial cortex both stimulates profilin-mediated actin polymerization by binding the diaphanous (Dia) formin-homology proteins and activates Rho-associated kinase, which phosphorylates the myosin regulatory light chain (*Drosophila* Spaghetti squash/Sqh), to promote myosin contractility. These events together trigger actomyosin filament assembly at the cleavage furrow and contractile ring formation (reviewed in refs 54, 55, 56).

In this screen, we identified 69 genes that had been either described as mitotic genes in FlyBase or identified in two previous screens for mitotic genes^57^,^58^. Knockdown of these 69 genes resulted in GSC loss (Figs 2 and 3, and Supplementary Data 6 and 2). This group of genes included *sqh*, *pav, tum, dia*, *feo, pebble (pbl)* and *neb*. As knockdowns of these cytokinesis genes resulted in GSC loss, these factors are probably important for GSC maintenance. We further tested 16 of these genes in spermatogonial cells (using *Bag of marbles (Bam)-Gal4*) and found that the knockdown of 14 genes (14/16=88%) with *Bam-Gal4* caused polyploidy (Supplementary Data 6 and Fig. 3n,o,q), a phenotype usually associated with cytokinesis defects^56^, indicating that most of the 69 mitotic genes regulate cytokinesis. Knockdown of these genes in GSCs may block GSC cytokinesis, causing polyploidy, followed by elimination of the defective GSCs through cell death.

We further crossed 32 of the 69 genes to cell-type-specific Gal4s and found that 25 (25/32=78%) resulted in strong GSC loss with *Nos-Gal4* (GSCs and early germline cysts) and various phenotypes with *c587-Gal4* (CySCs and early cyst cells) (Supplementary Data 6 and Fig. 3). We also generated *SMC2* (*Structural maintenance of chromosomes 2*) mutant mosaic clones of GSCs and found that SMC2 is required for GSC maintenance (Fig. 3p,q). These data together suggest that most of this group of genes intrinsically regulate GSC maintenance. We also identified 27 mitotic genes whose knockdowns resulted in GSC differentiation defects (Supplementary Data 2). These 27 genes are mostly ribosomal proteins or translational factors identified in the two previous screens for mitotic genes^57^,^58^; they may indirectly regulate mitosis by controlling the expression of key mitotic factors, as previously suggested^57^,^58^ or they may regulate GSC differentiation through mitosis-independent functions.

In a genome-wide RNAi screen focusing on *Drosophila* midgut ISCs^37^, we previously identified eight genes that regulate the mitotic cell cycle or mitotic cytokinesis. However, knockdown of these genes blocked mitotic cell division and disrupted the diploid status of ISCs, resulting in ISCs with excessive cell growth and large polyploid nuclei. It will be interesting to examine why disruption of the mitotic cell cycle resulted in GSC loss but excessive growth and large nuclei in ISCs. Notably, there were many more mitotic genes identified in the male GSC screen than in screens of ISCs, female GSCs and neuroblast^35^,^36^,^37^, suggesting that the mitotic genes play a more important role in male GSC regulation.

### Dlg-Scrib-EGFR and Toll pathways

Our screen identified two cell polarity complex components *discs large* (*dlg*), *scribbled* (*scrib)*, four EGFR signalling pathway components (*krn/Keren*, *Lk6*/*Lk6 kinase*, *Mkp3/Mitogen-activated protein kinase phosphatase 3* and *vav)* and two Toll signalling pathway components (*Nedd8/Neural precursor cell expressed, developmentally downregulated 8* and *dl/dorsal*). Knockdowns of these eight genes all resulted in GSC loss (Supplementary Data 2). The tumour suppressor genes *dlg* and *scrib* encode septate junction proteins with functions in epithelial cell polarity. Dlg is required for GSC and CySC maintenance by establishing and maintaining the male stem cell niche in somatic cells^59^,^60^. Scrib and the cell polarity complex may participate in the process involving Dlg. As described in a later section, EGFR signalling mainly functions in somatic cells to regulate GSC abscission^3^. The four EGFR-signalling-related components may affect GSC maintenance by negatively regulating EGFR signalling. The Toll pathway's function in GSC regulation is novel, therefore; it will be interesting to examine how this pathway regulates GSC self-renewal or maintenance.

In addition to the genes described above, we also identified 218 other genes (Supplementary Data 2) whose RNAi-mediated knockdowns resulted in GSC loss. These 218 genes include several genes that function in nucleosome, nucleoporin, chromatin binding and epigenetic modification (Supplementary Data 2), but most of the remaining genes' functions in GSC regulation are new. These genes provide a rich resource for future studies on GSC self-renewal and maintenance.

### Genes regulate GSC expansion or differentiation defects

RNAi-mediated knockdowns of genes required for GSC differentiation or of inhibitors of GSC self-renewal resulted in GSC differentiation defects or expansion. For simplicity, we will call them differentiation defects from now on. We identified 230 genes whose knockdown resulted in GSC differentiation defects (Figs 1c and 2f-h, and Supplementary Data 2).

### The EGFR-Raf-MAPK and Tor pathways

The EGFR-Raf-MAPK pathway was the first pathway reported to regulate GSC differentiation^24^,^25^. The germ cells associated with somatic cells mutant for the *Raf* and *Egfr* genes fail to differentiate and accumulate as early-stage germ cells, suggesting that the EGFR-Raf-MAPK pathway in somatic cells is required for GSC differentiation^24^,^25^. In addition, germ cells are known to release the EGFR ligand Spitz, to induce the somatic cell EGFR-Rac pathway, which regulates the germ cell enclosure, by somatic cells^61^. In somatic cells, Rac1 is positively regulated by the guanine nucleotide exchange factor Vav and negatively regulated by the small GTPase Rho1 (ref. 61). The encystment of GSCs by CySCs promotes abscission during GSC division after a regulated block in cytokinesis^3^. In this screen, we identified nine positive regulators (CG9375/Ras85D (Ras oncogene at 85D), CG10491/vn (vein), CG10334/spi, CG15793/Dsor1 (Downstream of raf1), CG1044/dos (daughter of sevenless), CG4063/ebi, CG5053/RASSF8, CG3403/Mob4 and CG10754) in the EGFR-Raf-MAPK pathway whose knockdown resulted in GSC differentiation defects.

Our screen also identified two components (*raptor* and *Tor*) in the Tor (target of rapamycin) signalling pathway. Knockdowns of these two genes resulted in GSC expansion or differentiation defects (Supplementary Data 2). The Tor pathway's function in GSC regulation is novel. It will be interesting to examine how this pathway regulates differentiation.

### Other genes involved in GSC differentiation

Besides the genes in the EGFR and Tor signalling pathways, and the 27 mitotic genes described above, we also identified 5 microtubule motor complex components, 9 mediator complex components, 11 components involved in vesicle transport/secretion, 5 nucleoporin components and 162 other genes. These genes will be a rich resource for future studies on GSC differentiation.

### Signal from local niche and distant organs regulate GSC fate

To investigate the cell-type specificity of the above-identified genes, we knocked down 113 genes by crossing their corresponding RNAis with both *Nos-Gal4* and *c587-Gal4* (Supplementary Data 6). Among the 113 lines that resulted in strong abnormal GSC phenotypes with *Act-Gal4*, only 43 lines (38%) had abnormal and similar GSC phenotypes with *Nos-Gal4* (Supplementary Data 6), suggesting that more than half of the genes (62%) identified in the screen regulate GSC fates non-autonomously. Of the 113 lines, 74 (65%) had abnormal (either gain or loss) GSC phenotypes with *c587-Gal4*, suggesting that CySCs or early cyst cells are important in regulating GSC fates. In addition, 73 (65%) of the 113 lines had abnormal GSC phenotypes with *Nos-Gal4* and *c587-Gal4*, similar to their phenotypes with *Act-Gal4*, suggesting that 65% of the genes regulate GSC fates intrinsically or externally from CySCs or local early cyst cells, and the other 35% regulate GSCs from distant organs. We also found that 28 of the 113 genes had no phenotypes with both *Nos-Gal4* and *c587-Gal4*, suggesting that these genes regulate GSCs outside of GSCs and CySCs. We selectively crossed the RNAi lines of these 28 genes with *Upd-Gal4* (hub cells) and *Elav-Gal4* (pan neurons). None of the 10 lines with *Upd-Gal4* had GSC defects, whereas 9 of the 23 lines with *Elav-Gal4* did have defects, indicating that these 9 genes regulate GSCs from neurons and 14 genes regulate GSCs from other organs.

### *Tctp* differentially regulates GSCs from different cells

We further characterized CG4800, a *Drosophila* translationally controlled tumour protein gene (*Tctp*). Its product was initially identified as a guanyl-nucleotide exchange factor for *Drosophila* Rheb GTPase, essential for growth and proliferation^62^, and was recently characterized as a regulator of ataxia telangiectasia-mutated kinase activity to control genome stability and organ development in *Drosophila*^63^. *Tctp* knockdown with *Act-Gal4*, *Nos-Gal4* and *c587-Gal4* resulted in GSC differentiation defects, GSC loss and GSC differentiation defects, respectively (Fig. 4a–c). We further generated mosaic clones with mutant *Tctp* and found that *Tctp* is required for GSC maintenance (Fig. 4d–g). Therefore, *Tctp* is required in GSCs for GSC maintenance and in CySCs for GSC differentiation.

### Vers has multiple roles in regulating testis stem cells

**Figure 6:**
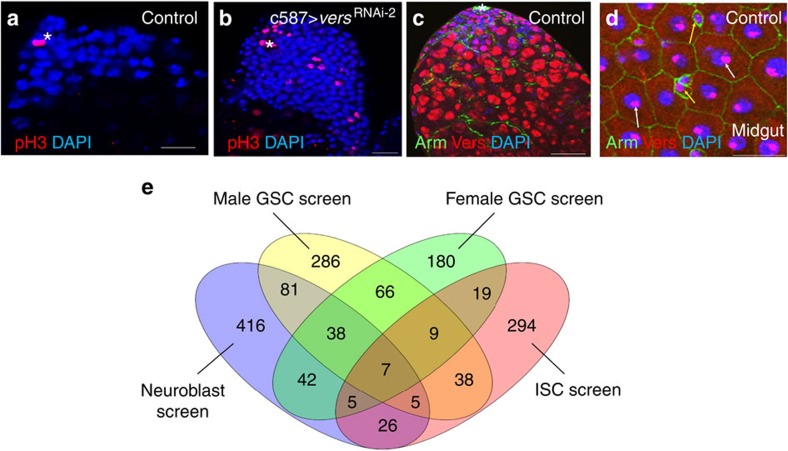
Cell proliferation in *vers* knockdown and Vers expression. (**a**) Wild-type (control) testis. (**b**) Knockdown of *vers* in the CySC lineage (*c587-Gal4*) resulted in an expansion of mitotic cells. Vers expression in the testis (**c**) and in the midgut (**d**). (**e**) Comparison of male GSC, ISC, neuroblast and female GSC RNAi screens. Number of genes identified in the male GSC, ISC, neuroblast and female GSC screens. Seven genes were found in all four screens; 9 genes were shared by male GSC, female GSC and ISC screens; and 38 genes were shared by male GSC, female GSC and neuroblast screens. Venn diagram was prepared from: http://bioinfogp.cnb.csic.es/tools/venny/index.html. Testes of the indicated flies were dissected, stained with the antibodies to phospho-Histone 3 (pH3) (red, marks mitotic cells) (**a**,**b**), Vers (red, all type of cells in the testis and midgut), Arm (green, hub cells in testis (**c**) and epithelial membrane in the midgut (**d**)) and DAPI (blue, marks the nuclei), and analysed by confocal microscopy. Asterisks indicate hub cells. Yellow arrows in **d** indicate ISC/EB (enteroblast) cells and white arrows indicate enterocyte (EC) cells. The flies were cultured at 29 °C for 7 days in **a**–**d**. Scale bars, 10 μm.

Heterochromatin protein 1 has several conserved roles, including gene silencing transcription, DNA replication and DNA repair^51^. In this screen, we identified *l(3)j2D3* (*CG6801*, *lethal (3) j2D3*), which encodes a component of the heterochromatin protein 1 protein complex. We renamed the gene ‘*versatile*' (*vers*) based on its versatile functions described below. *vers* knockdown by transgenic RNAi with an *Act-Gal4* driver resulted in GSC differentiation defects (Supplementary Data 2). To examine the function of *vers* in the germline versus the soma, we knocked down *vers* using cell-type-specific Gal4s and two independently generated *UAS-vers*^RNAi^ transgenic fly lines. Depleting *vers* in the CySC lineage (*c587>vers*^RNAi^; Fig. 5 and Supplementary Figs 1 and 2) caused GSC differentiation defects (Fig. 5a,b). We further examined CySC proliferation and changes in *c587>vers*^RNAi^ flies by staining with antibodies to Zfh1 (marks CySCs and their immediate cyst cell daughters; Fig. 5c,d), phospho-Histone 3 (Fig. 6a,b) and Tj (traffic jam, a transcription factor expressed in CySCs, early cyst cells and hub cells; Supplementary Fig. 2b,c). Compared with wild-type testes, the numbers of Zfh1-positive, p-H3-positive and Tj-positive cells were either dramatically increased in the testes of *c587>vers*^RNAi^ flies, suggesting that *vers* knockdown caused a dramatic expansion of CySCs and GSC differentiation defects. We also stained the *c587>vers*^RNAi^ flies with antibodies to Cic (capicua, a transcriptional repressor downregulated by EGFR signalling)^64^ and Mad (Mothers against dpp, a transcriptional factor in the Dpp signalling pathway). Compared with wild-type testes, the numbers Cic-positive and Mad-positive cells were dramatically increased in the testes of *c587>vers*^RNAi^ flies (Fig. 5e,f), suggesting that *vers* knockdown caused the downregulation of EGFR signalling and the activation of Dpp signalling. We made an anti-Vers antibody, stained the testes and midguts of adult *Drosophila*, and found that Vers is ubiquitously expressed and localized to the nucleolus (Fig. 6c,d).

We found that depleting *vers* in the germ cell lineage using *Nos-Gal4* resulted in a significant decrease (Student's *t*-test, *P*<0.05) in the number of GSCs associated with the hub compared with the wild-type control (Supplementary Fig. 1a–d). To examine the function of *vers* in GSCs, we generated negatively marked GSC clones of wild-type or *vers*^j2D3^ flies^65^ using the FLP (flippase)/FRT (flippase recognition target) mosaic analysis technique^66^. Testes with LacZ (*arm-lacZ*)-negative clones were counted 2 and 7 days after clone induction (ACI). As expected, the *FRT*^80B^ control testes contained many LacZ-negative GSCs and their differentiated progenies (Supplementary Fig. 3a,b,e) at 2 and 7 days ACI. The *FRT*^80B^-*vers*^j2D3^ testes at 2 days ACI also contained LacZ-negative GSCs and their differentiated progenies (Supplementary Fig. 3c,e). However, at 7 days ACI, LacZ-negative *vers* homozygous mutant GSCs had recovered at negligible levels (Supplementary Fig. 3d,e). Staining testes containing *vers* mosaic clones with anti-Stat92E antibodies revealed similar levels of Stat protein in the wild-type and *vers*-mutant GSCs (Supplementary Fig. 2a), indicating that Stat signalling is not required for Vers's function in GSCs.

These data together suggest that the heterochromatin protein Vers has multiple roles in regulating testis stem cells. It regulates GSC maintenance through a Stat-independent pathway in GSCs and regulates GSC differentiation and CySC expansion, possibly by upregulating the EGFR signalling pathway and/or downregulating the Dpp signalling pathway in somatic cells.

### Comparing our screen to previous stem cell screens

We compared our results with those of previous screens focused on neuroblast^35^, female GSCs^36^ and ISCs^37^, to identify both common and unique factors regulating the self-renewal and differentiation of different stem cell systems (Fig. 6e). Among the 530 genes identified in the male GSC screen, 7 were identified in all four screens, 12 were identified in the neuroblast, ISC and male GSC screens, and 45 were identified in the neuroblast, female GSC and male GSC screens (Fig. 6e and Supplementary Data 4). Most of the shared genes are regulators of basic, intrinsic cellular processes, such as messenger RNA splicing, chromosome assembly and disassembly, the cytoskeleton, the mitotic chromosome and cytokinesis (Supplementary Data 4). Of the 530 genes, 286 (54%) were male GSC-specific genes and were likely to include external regulators from both the local environment and distant organs. Future characterization of the common stem cell intrinsic regulators will increase our understanding of basic stem cell biology and further study of the external regulators will help reveal how stem cell behaviours are modulated by both the local microenvironment and signals from distant organs.

## Discussion

*Drosophila* male GSCs provide an excellent genetic system for studying niche-stem cell interactions. To comprehensively identify genes and pathways that regulate GSC fates in the whole organism, we performed a genome-wide transgenic RNAi screen through the ubiquitous knockdown of genes in adult *Drosophila* and identified 530 genes that regulate various fates of adult *Drosophila* testis GSCs.

As we screened the RNAi lines eliciting an adult lethal phenotype, we might have missed some adult testis-specific genes in this screen. A number of published genes that function in testis stem-cell regulation were missing from the genes identified in our screen, including *zfh1* (ref. 22), *chinmo*^21^, genes in the Hh (hedgehog) signal transduction pathway^23^,^67^, *baz* and *par-1* (ref. 30). In this screen, we focused only on genes whose RNAi-mediated knockdown affected GSC numbers. Loss-of-function of the above genes does not affect the GSC numbers; therefore, our screen did not identify them.

By analysing the genes identified here, we reached a number of important conclusions. First, more than half the genes identified in our screen regulate GSC fates non-autonomously. Second, both the local microenvironment and signals from distant organs regulate GSCs. Third, the characterization of Tctp and Vers showed that the same protein could regulate GSC fates differentially in different cell types and through multiple pathways. Fourth, mitosis/cytokinesis proteins are especially important for male GSC maintenance. We identified 69 mitosis/cytokinesis genes whose RNAi-mediated knockdown resulted in a strong GSC loss phenotype. In our previous screen for *Drosophila* ISCs, we identified only eight mitotic genes^26^. In previous genome-wide RNAi screens for neuroblast and female GSCs, only a few mitosis/cytokinesis genes were identified^35^,^36^,^37^. Collectively, these findings suggest that mitosis/cytokinesis genes are more important in GSC regulation.

To better analyse our results, we generated a gene interaction network by querying publicly available databases containing yeast–two-hybrid interactions, protein–protein interactions, text-mining data and genetic interactions between *Drosophila* genes (Supplementary Figs 4 and 5). We performed a complex-enrichment analysis using COMPLEAT^68^ and identified a number of protein complexes required for GSC fate determination (Supplementary Fig. 4). However, as we have not assigned our 530 genes to GSCs, CySCs or other cell types and the protein interaction network is only meaningful in the same cells, these data may only serve as an indirect reference.

To our knowledge, this is the first genome-wide gene-knockdown screen performed in a whole animal, to identify systemic regulators of stem cells. Stem cells are regulated both intrinsically and externally by signals from the local microenvironment^39^ and distant organs^40^,^41^. Thus, it is difficult to fully appreciate the complexity of stem cell regulation if we limit our screen to intrinsic regulators. By performing a genome-wide transgenic RNAi screen by the ubiquitous knockdown of genes with *Act-Gal4*, we identified intrinsic regulators, as well as regulators from the local microenvironment and distant organs. Further characterization of these genes by knocking them down with cell-type-specific Gal4s will lead to a better understanding of GSC regulation in the whole animal. Our findings provide valuable insight and resources for studying stem cell regulation at the organismal level.

## Methods

### Fly stocks

Oregon R or *UAS-lacZ*^RNAi^ were used as wild type. RNAi stocks used in this study were: *tum*^RNAi^ (BDSC number 35007 [BL35007]), *Nedd8*^RNAi^ (BL33881), *bab2*^RNAi^ (VDRC Transformant ID 49042 [v49042]), *Taf8*^RNAi^ (v27870), *Cka*^RNAi^ (BL34522), *vn*^RNAi^ (v50538), *SMC2*^RNAi^ (BL32369), *APC10*^RNAi^ (BL34858), *Cenp-C*^RNAi^ (BL34692), *Tctp*^RNAi^ (BL32911), *vers*^RNAi-1^ was original *l(3)j2D3*^RNAi-1^ (v22439) and *vers*^RNAi-2^ (v101038). *FRT*^42D^*SMC2*^f06842^ and *FRT*^8oB^*vers*^j2D3^ were obtained from Kyoto Stock Center. *FRT*^82B^*Tctp*^h5p^ was provided by Dr Kwang-Wook Choi, Korea Advanced Institute of Science and Technology.

The following Gal4 alleles were used to drive UAS lines: *Nos-Gal4* (*nanos-Gal4VP16*)^69^, *Act-Gal4* and *Elav-Gal4*, obtained from the BDSC; *upd-Gal4* and *c587-Gal4*, provided by Dr Ting Xie, Stowers Institute for Medical Research; and *Bam-Gal4*, provided by Dr Xin Chen, Johns Hopkins University. Flies were raised on standard fly food at 25 °C and 65% humidity, unless otherwise indicated.

### RNAi stocks used in the screen

*UAS-RNAi* lines were generated by VDRC and the Transgenic RNAi Project, and are available at VDRC and the BDSC. The sequences used for VDRC knockdown strains are available for each line at https://stockcenter.vdrc.at and for BDSC knockdown strains are available for each line at http://flystocks.bio.indiana.edu.

### Generation of mutant GSC clones

Clones of mutant GSCs were generated as previously described^6^. To generate *SMC2*-mutant GSC clones, *FRT*^42D^*+* and *FRT*^42D^*SMC2*^f06842^/*Cyo* virgin females were mated with males of genotype *FRT*^42D^*arm-lacZ*/*Cyo*; *MKRS, hs-flp/+.* To generate *Tctp*-mutant GSC clones, *FRT*^82B^*+* and *FRT*^82B^*Tctp*^h5p^/*TM3, Sb* virgin females were mated with males of genotype *SM6, hs-flp/+; FRT*^82B^*arm-lacZ*/*TM3, Sb*. To generate *vers*-mutant GSC clones, *FRT*^80B^*+* and *FRT*^8oB^*vers*^j2D3^/*TM3, Sb* virgin females were mated with males of genotype *SM6, hs-flp/+; FRT*^80B^*arm-lacZ*/*TM3, Sb*. One- or 2-day-old adult males carrying an *arm-lacZ* transgene in *trans* to the mutant-bearing chromosome were heat shocked four times at 37 °C for 1 h, at intervals of 8–12 h. The males were transferred to fresh food every day at 25 °C. The testes were removed 1, 2 or 7 days after the first heat-shock treatment and processed for antibody staining.

### RNAi-mediated gene depletion

Male *UAS-RNAi* transgenic flies were crossed with virgin female of genotype *Act-Gal4; tub-Gal80*^ts^ (*Act*^ts^), *Nos-Gal4*, *upd-Gal4* or *c587-Gal4; tub-Gal80*^ts^ (*c587*^ts^). The flies were cultured at 18 °C. Three- to 5-day-old adult flies with the appropriate genotype were transferred to new vials at 29 °C for 7 days before dissection.

### Immunofluorescence staining and microscopy

For immunofluorescence staining, testes were dissected in 1 × PBS, then transferred to 4% formaldehyde in 1 × PBS and fixed for 30 min. The testes were then washed three times in 1 × PBST (PBS containing 0.1% Triton X-100) for 2 min each, then blocked with 5% goat serum in 1 × PBST for 1 h. Samples were then incubated with primary antibody in PBST at 4 °C overnight. Samples were then washed for 30 min (three 10 min washes) in 1 × PBST, incubated with secondary antibody in 1 × PBST at room temperature for 2 h, washed for 30 min (three 10 min washes), then rinsed in 1 × PBS (twice, 2 min each) and mounted in VECTASHIELD with DAPI (4,6-diamidino-2-phenylindole; Vector Labs).

Confocal images were obtained using a Zeiss LSM510 system and were processed with Adobe Photoshop 7.0. GSCs were scored as Vasa-positive cells adjacent to the hub (detected using Fas 3) and containing dot spectrosome (detected using 1B1). Only images with a clear view of the complete hub were used.

The following antisera were used: rabbit polyclonal anti-Vasa antibody (1:5000; gift from R. Lehmann), rabbit polyclonal anti-β-Gal (β-galactosidase) antibody (1:1,000; Cappel), mouse monoclonal anti-β-Gal antibody (1:100; Invitrogen), mouse monoclonal anti-Hts antibody 1B1 (1:4; Developmental Studies Hybridoma Bank (DSHB)), mouse monoclonal anti-Fas3 antibody (1:10; DSHB), rabbit polyclonal anti-green fluorescent protein (GFP) antibody (1:200; Molecular Probes), mouse monoclonal anti-GFP antibody (1:100; Invitrogen), rabbit polyclonal anti-Thr3-phosphorylated histone H3 antibody (1:200; Upstate), guinea pig polyclonal anti-Zfh1 (1:2000; gift from Dr James B. Skeath, Washington University in St Louis), rabbit polyclonal anti-Cic antibody (1:1,000; gift from Dr Iswar Hariharan, University of California, Berkeley) and rabbit polyclonal anti-Smad3 antibody (1:1,000; Cell Signaling). Rabbit polyclonal anti-Stat92E antibody (1:1,000), rabbit polyclonal anti-Vers/l(3)j2D3 antibody (1:1,000) and guinea pig polyclonal anti-Tj antibody were generated in our laboratory. Secondary antibodies were goat anti-mouse, goat anti-guinea pig and goat anti-rabbit IgG conjugated to Alexa 488 or Alexa 568 (1:400; Molecular Probes). DAPI (Molecular Probes) was used to stain DNA.

### Quantitative PCR

Total RNA from Act>RNAi adult fly testes was isolated using the RNeasy Mini Kit (Qiagen) with on-column DNase digestion, to remove genomic DNA. Complementary DNA was synthesized using the ThermoScript RT-PCR System (Invitrogen). Real-time PCR analysis was performed on a real-time PCR system, Mastercycler Realplex (Eppendorf), using SYBR Green PCR Master Mix (Clontech). Primers were selected using FlyPrimer Bank. The quantitative PCR primers used are shown in Supplementary Table 1.

### Bioinformatics analysis

The GSC genetic network (Supplementary Fig. 5) was generated using STRING 10.0. Complex analysis (Supplementary Fig. 4) was done using COMPLEAT (http://www.flyrnai.org/compleat/), a tool that annotates protein complexes from both literature and predictions from protein–protein network, and does gene-set enrichment analysis based on protein complexes.

### Statistical analysis

Statistical analyses of GSC and CySC numbers (mean±s.e.m.) were performed using GraphPad Prism programme. Sample sizes (*n*) reported reflect the individual testis number. *P*-values were obtained between two groups using the Student's *t*-test. For all statistical analysis, differences were considered to be statistically significant at values of *P*<0.05.

### Data availability

The authors declare that the data supporting the findings of this study are available within the article and its Supplementary Information files, and from the corresponding author upon reasonable request.

## Additional information

**How to cite this article:** Liu, Y. *et al.* Whole-animal genome-wide RNAi screen identifies networks regulating male germline stem cells in *Drosophila*. *Nat. Commun.* 7:12149 doi: 10.1038/ncomms12149 (2016).

## Supplementary Material

Supplementary Information Supplementary Figures 1-5, Supplementary Table 1

Supplementary Data 1 Total RNAi lines screened.

Supplementary Data 2 Male GSC screen results.

Supplementary Data 3 Confidence analysis.

Supplementary Data 4 Comparison of Nb, ISC, female GSC, and male GSC screens.

Supplementary Data 5 RNAi efficiency.

Supplementary Data 6 Knockdowns of 113 selected genes using cell-type-specific Gal4.
