# Supplementary material for: Whole-animal genome-wide RNAi screen identifies networks regulating male germline stem cells in Drosophila
Source: Nat Commun. 2016 Aug 3;7:12149. doi: 10.1038/ncomms12149 (PMC4976209; doi:10.1038/ncomms12149)
Supplement: Supplementary Information — Supplementary Figures 1-5, Supplementary Table 1 [file ncomms12149-s1.pdf]

Supplementary information

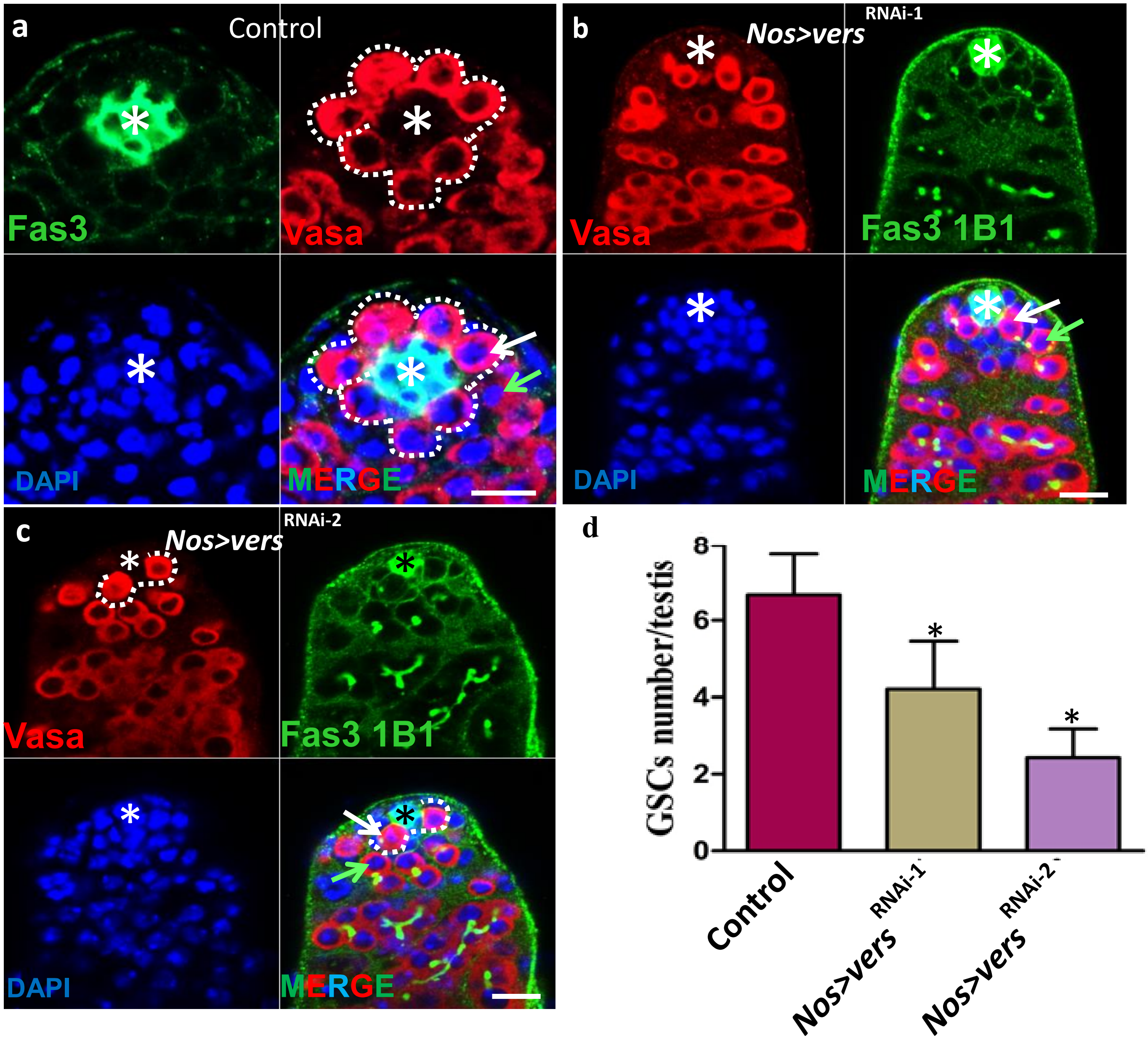

Supplementary Figure 1 | Vers regulates GSC maintenance at the niche. (**a**) Wild-type (control) testis. (**b,c**) Knockdown of *vers* in germ cell lineage (*Nos-Gal4*) resulted in a decrease in the number of GSCs. (**d**) Quantitative analysis of the GSC number in wild-type (control) and *vers*-knockdown testes. All values are mean±S.D. Statistical significance determined by Student's t-test, *P<0.05. Testes of the indicated flies were dissected, stained with antibodies to Vasa (red, marks all germ cells including GSCs, red color), Fas3 (green, hub cells at the apex), 1B1 (green, marks round spectrosomes and branched fusomes) and DAPI (blue, marks the nuclei), and analyzed by confocal microscopy. The flies were cultured at 29°C for 7 days in (**a**–**c**). White arrows near hub cells indicate GSC and green arrows indicate GBs (in **a-c**). Asterisks indicate hub cells. Scale bars: 10 μm.

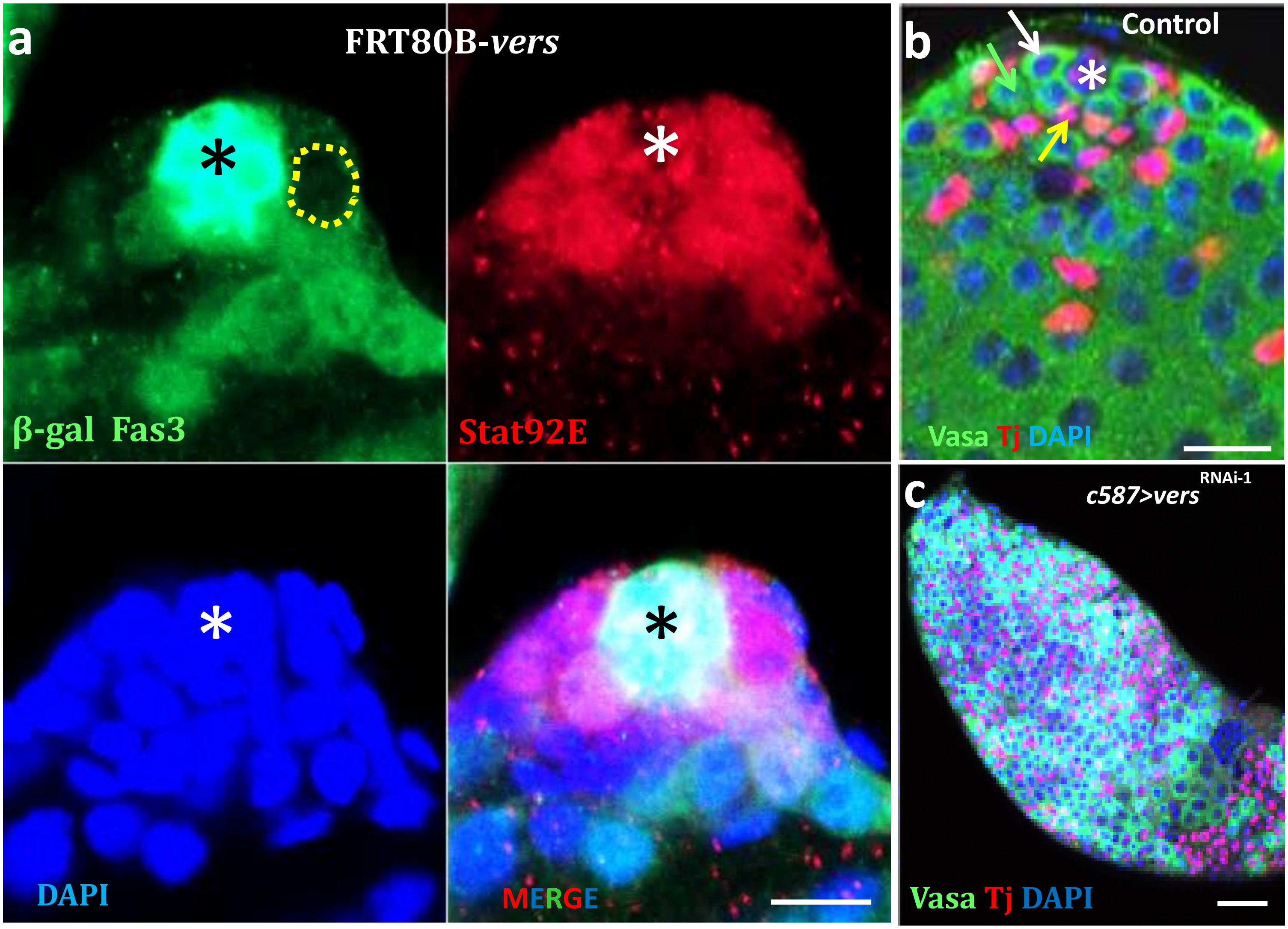

Supplementary Figure 2 | Stat signaling is not required for *vers*'s function in GSCs. (**a**) *FRT*^{80B}- *vers* mosaic clones of GSCs 2 days ACI. The Stat protein level in the *vers*-mutant clone was normal. (b) Wild-type (control) testis. (**c**) Knockdown of *vers* in CySC (*c587-Gal4*) dramatically increased the Tj (red) expression. The testes of the indicated flies were dissected, stained with antibodies to β-galactosidase (green), Fas3 (red, hub cells at the apex), and DAPI (blue) stains nuclei in (**a**); Vasa (green, marks all germ cells including GSCs), Tj (red, marks CySCs, early cyst cells and hub cells) and DAPI (blue, marks the nuclei) in **b,c**, and analyzed by confocal microscopy. The flies were cultured at 29°C for 7 days in (**b, c**). White arrow near hub cells indicates GSC, green arrow indicates GBs, and yellow arrow indicates CySCs (in **b**). Asterisks indicate hub cells. Scale bars: 10 µm.

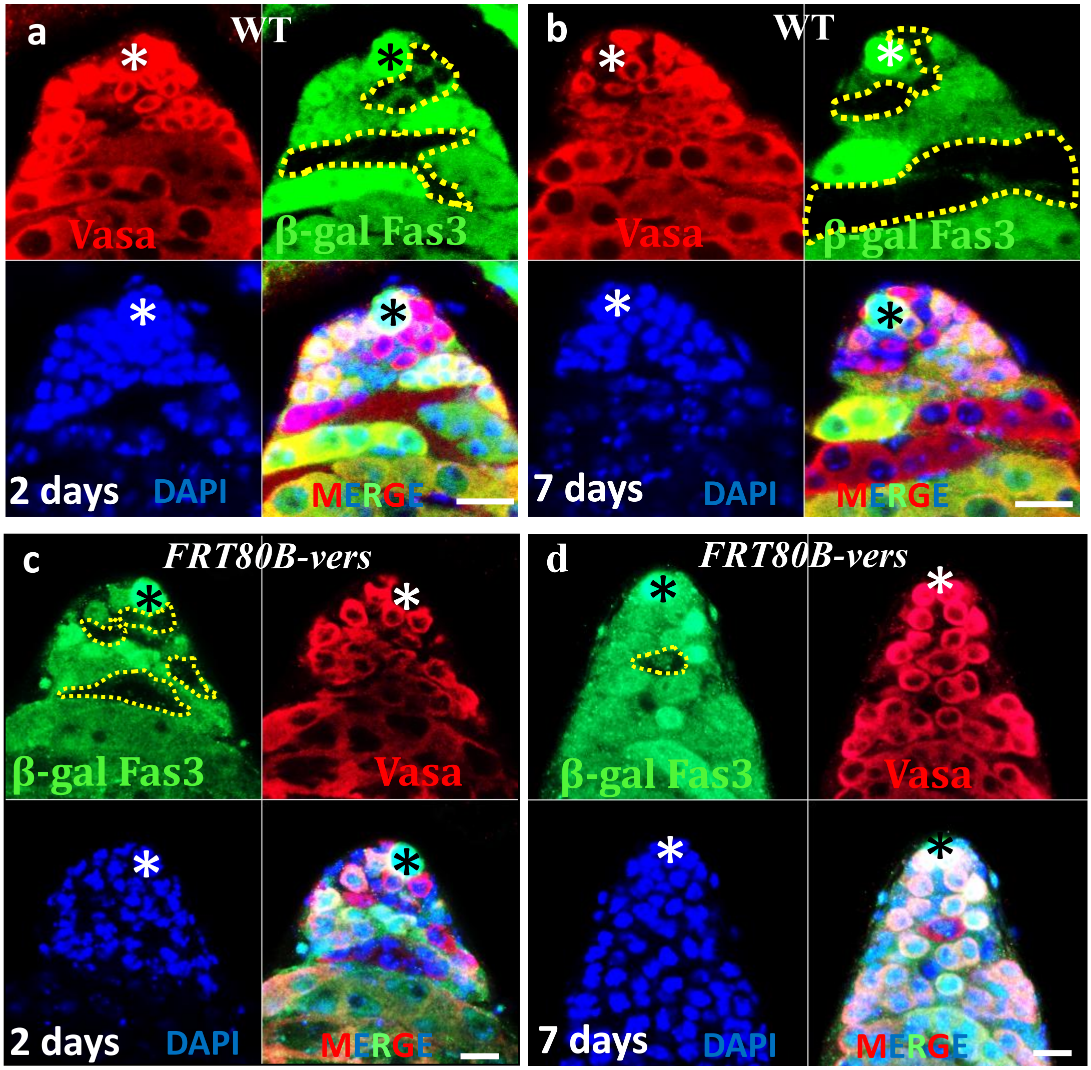

e

Genotype	2 days ACI	7 Days ACI
Testis with marked GSCs		
WT (*FRT80B*)	33/37 (89.19%)	51/64 (79.69%)
FRT80B-vers	44/62 (70.97%)	3/45(6.67%)

Supplementary Figure 3 | *vers* is intrinsically required for GSC maintenance. (**a, b**) *FRT*^{80B}-*piM* mosaic clones of GSCs 2 days (**a**) and 7 days (b) ACI. (**c, d**) *FRT*^{80B}- *vers*^{j2D3} mosaic clones of GSCs 2 days (c) and 7 days (**d**) ACI. (**e**) Quantitative analysis of testis with Arm-lacZ-negative GSC clones in wild-type (WT) and *vers* mutant flies 2 days and 7 days ACI. The testes of the indicated flies (**a-d**) were dissected, stained Vasa (red, germ cells including GSCS), β-galactosidase (green), and DAPI (blue marks the nuclei), and analyzed by confocal microscopy. Yellow dotted lines indicate GSCs clones. Asterisks indicate hub cells. Scale bars: 10 μm.

Protein complexes

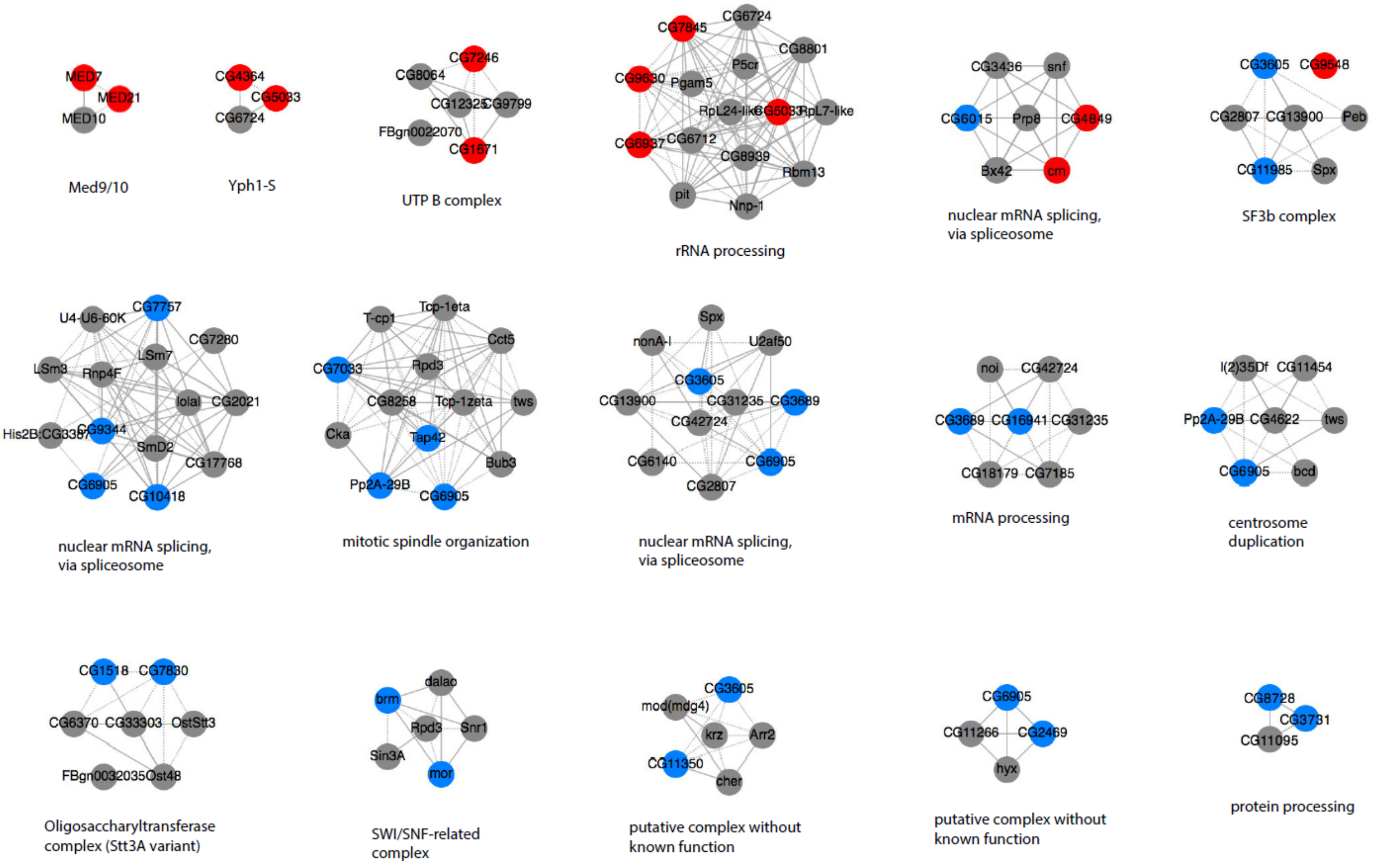

Supplementary Figure 4 | Representative protein complexes identified using COMPLEAT. Red and blue genes were identified in the screen. Grey genes were not identified in the screen but were identified by querying publicly available databases. The full list of protein complexes is shown in Supplementary Table 1 and Supplementary Table 3.

Regulatory Network

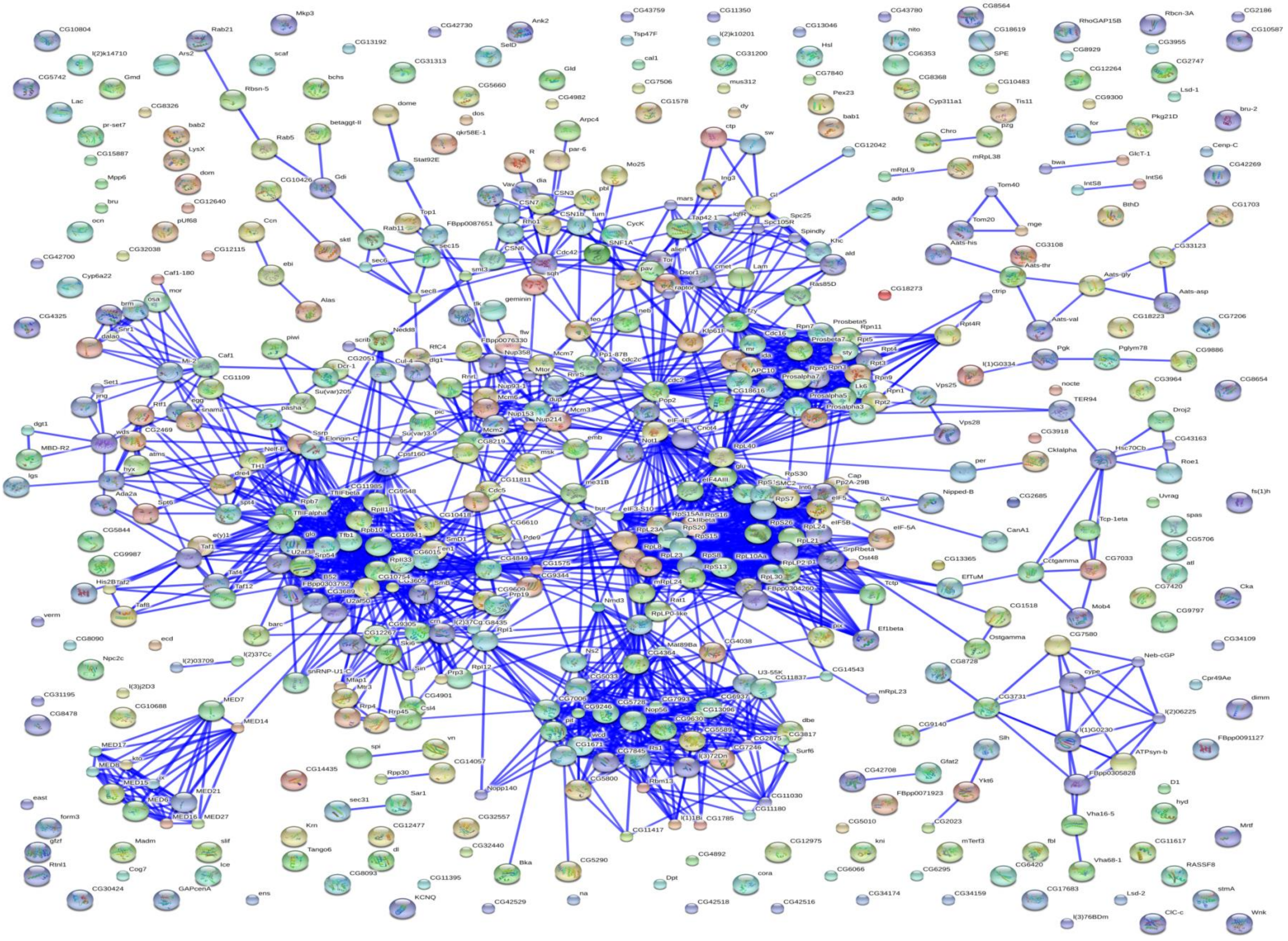

Supplementary Figure 5 | Regulatory network for genes identified in the GSC screen.

Supplementary Table 1. **qPCR primers used**

Gene	RNAi line	Forward	Reverse
CG4817/ssrp	v44343	GGAGTCTTGTGTTCCGGACG	AAACTTGCCCAGCTTCTCGT
CG16916/Rpt3	v100681	AATCGATTGCGGACAGGGTT	GTCGGAGAGCTCGTCCTTTT
CG10804	v100400	TATAGTGGGGGCACTCTGCT	GGGCGATAGGTACTTCCAGC
CG13223	v1684	TGCCCGCATTCAGAAAATGG	CTTCACCAGGAAGGTTCCCC
CG9327/Prosα3	v104373	AATGGCTCGCCGCTATGATT	CGAGCAGACCATGTTGTCGT
CG8553/SelD	v35959	ACAATCGCACAAGCAAACCTAT	ACCAGCTTGGAGAGCACATC
CG4797	v10598	TCCAGGAAGCTCTCCACCAT	TGGACACTTGCTATCCAGGAG
CG15304/Neb-cGP	v107937	CCGTGCAAATGTGGCCAAGG	AATGCACTTCGTGGGTACAGT
CG5999	v33339	CCCTGGAGGTTTCCTATGCC	CGTTGCCATAAACTTCCTTGT
CG18332/CSN3	v12821	TCGTCGAAGGCAAGATAGCG	GGTTTTAGTGAGCCGCTGGA
CG7257/Rpt4R	v22548	ACGCTGAAATCGGTGGACTT	TGCAGAGGAAACGACCTTCA
CG5650/pp1-87B	v35024	ATGCATGAAACCCGGGGC	ATCTCAATGGGTGTGGGGTG
CG12042	v31623	CACTGGTGGCAGGAAAAAGC	GTGGGGACGAAGAAATGGGT
CG1152/Gld	v38041	CCTCAGCCTGCGATTGTTTG	GCTCCGATCGAAACCGACTG
CG14656/Ctrip	v48117	GTGAACTTGGTGCTTCGGATG	GTTTCCTACGGATGAAGCCGA
CG32848/pde9	v1490	ATCCGGACAAGGTCAGCAAG	TGAGGCGCCTGTAGTACTCT
CG6998/Ctp	v43115	GAACCGCTAAAAAGGGCCAC	TGTAGGCCGCAATGTCCTTT
CG1768/dia	BL33424	TGGACGTGCTGGGCAAAAT	CGTACCCGGACATTGTCGTT
CG8648/Fen1	BL35764	TTTTTCGGTCGCAAGGTAGC	CCGGACTTGAGATCTGGTGG
CG4206/Mcm3	BL34686	AGCGTTCACTATTGCCCCAA	GAATGGTCAGCGTCTGGTGA
CG31258/Cenp-C	BL34692	TCGAGATAGATAGGGGCACTAGG	ATGGCGTGGGTCTATCACCT
CG10938/Prosα5	v16105	ATTTGCACGCCAGAAGGAGT	GATCAGAGTCCTGGCATCGG
CG32183/Ccn	v38208	GAATCCGGCCAATTGCAGTG	TCGGCATGAAATCGTGTCCT
CG11450/Spc105R	BL36100	GCAGAAGTTCGCTGCGTAAG	TGTCCTCAGCATGGTGTTCC
CG14447/Grip	BL41978	TGCGCGTCGATAATGTCTCA	GTTCATCGGTCGCTCGATCT
CG10850/ida	BL34552	TCAGCATGGTGGGCGTTTAT	GCTACGGCCTCCACTTTCTT
